# Far infrared intervention on brain changes in patients with alcohol dependence: a pilot longitudinal MRI study

**DOI:** 10.3389/fpsyt.2026.1759791

**Published:** 2026-03-09

**Authors:** Yu Liu, Wenxiang Geng, Tao Wang, Yang Liu, Wenzheng Li, Zheng Chang, Xianjun Yang, Yanyan Chen, Qingrong Xia, Zenghui Ding

**Affiliations:** 1 Department of Addiction Medicine, Affiliated Psychological Hospital of Anhui Medical University, Hefei, Anhui, China; 2 Department of Addiction Medicine, Hefei Fourth People's Hospital, Hefei, Anhui, China; 3 School of Information and Artificial Intelligence, Anhui Agricultural University, Hefei, Anhui, China; 4 Hefei Institutes of Physical Science, Chinese Academy of Sciences, Hefei, Anhui, China; 5 School of Information Science and Technology, Hainan Normal University, Haikou, Hainan, China; 6 Science Island Branch, Graduate School of University of Science and Technology of China, Hefei, Anhui, China

**Keywords:** alcohol dependence, far infrared intervention, gray matter, MOCA, routine withdrawal, sMRI, VBM

## Abstract

**Objective:**

Chronic alcohol consumption leads to a range of neuropathological changes. Far-infrared (FIR) therapy is a non-invasive thermal intervention that may influence systemic hemodynamics and recovery-related processes; however, its potential role in neurological recovery in alcohol dependence remains insufficiently characterized.

**Methods:**

Male patients were recruited and divided into a conventional withdrawal group and an FIR intervention group. Researchers collected structural MRI (sMRI) data before and after the intervention, using Voxel-based morphometry (VBM) to analyze whole-brain gray matter volume. The Montreal Cognitive Assessment (MoCA) was also administered.

**Results:**

conventional withdrawal alone enhanced brain regions linked to cognition and sensorimotor function, such as the cingulate gyrus and hippocampus. The FIR group, however, showed additional increases in gray matter volume in key areas like the prefrontal cortex, cerebellum, and insula. On MoCA tests, the FIR group consistently scored higher, with differences at baseline and after intervention approaching statistical significance.

**Conclusion:**

These findings suggest that while abstinence promotes brain reorganization, FIR therapy may provide additional support for neuroplasticity and cognitive stabilization, aiding in the structural recovery of key brain regions affected by Alcohol dependence. This study is preliminary in nature due to the modest sample size and the non-randomized design. Larger randomized controlled studies with longer follow-up are required to confirm the robustness and clinical significance of the observed effects.

## 1 Introduction

Alcohol dependence is a chronic brain disease characterized by frequent relapse and complex pathological mechanisms involving genetic, environmental, psychological, and biological factors (1). It significantly impairs behavioral decision-making, emotional regulation, and brain structure and function. Neuroimaging studies have identified structural and functional abnormalities in key brain regions, including the prefrontal cortex, hippocampus, and basal ganglia, in individuals with Alcohol dependence (2). The prefrontal cortex governs decision-making, planning, and impulse control; the hippocampus plays a crucial role in memory formation and spatial navigation; and the basal ganglia regulate motor control and reward processing. Reduced gray matter volume in these regions is associated with cognitive impairments and deficits in impulse control (3). Although current treatments—such as medications, psychotherapy, and behavioral interventions—can alleviate some symptoms of Alcohol dependence, their efficacy remains limited, and relapse rates are high (4).

Far infrared (FIR) therapy has recently gained attention as a non-invasive modality for biological regulation and thermal therapy. The FIR spectrum encompasses wavelengths (typically 3–1000 μm) that interact with biological tissues differently than near-infrared (NIR) radiation. In this study, we utilized an FIR wavelength range of 8–10 μm. Unlike NIR, which can penetrate deeper into tissues, FIR energy in this range is primarily absorbed by water molecules in the superficial layers of the skin, generating a mild and uniform thermal effect without causing excessive surface heating (5, 6).

The potential neurorestorative effects of FIR in the context of Alcohol dependence are likely mediated through systemic physiological mechanisms rather than direct deep-brain stimulation. First, FIR induces a systemic hemodynamic effect. The absorption of FIR energy raises cutaneous temperature, triggering the release of vasoactive mediators such as nitric oxide (NO) and inducing systemic vasodilation (7, 8). This improvement in microcirculation can indirectly enhance cerebral blood flow (CBF) and perfusion, counteracting the cerebrovascular dysfunction and hypoperfusion often observed in chronic alcohol dependence.

Second, FIR therapy may exert neuroprotection through molecular anti-inflammatory mechanisms. Systemic thermal stress, such as that induced by FIR or sauna therapy, has been shown to upregulate heat shock proteins (HSPs) and reduce oxidative stress markers (9–11). Given that chronic alcohol consumption is associated with neuroinflammation and oxidative damage, the systemic anti-inflammatory environment promoted by FIR may provide a favorable milieu for neuroplasticity and structural brain recovery. Additionally, population-based studies have linked regular thermal therapy (sauna bathing) with a reduced risk of dementia and Alzheimer’s disease, further supporting the potential link between systemic thermal exposure and neuroprotection (12). However, systematic research on FIR-induced brain structural changes in alcohol-dependent patients remains limited.

Voxel-based morphometry (VBM) is a widely used neuroimaging technique for quantitatively assessing brain structural changes at the voxel level (13). Unlike region-of-interest (ROI)-based methods, which focus on predefined brain areas, VBM enables whole-brain analysis, making it particularly suited for studying widespread neuroanatomical alterations in Alcohol dependence. Previous VBM studies have consistently reported reduced gray matter volume in key cognitive and affective brain regions, such as the prefrontal cortex, insula, and anterior cingulate cortex, in individuals with Alcohol dependence (14, 15). These structural deficits are strongly associated with impairments in executive function, emotional processing, and impulse control. Longitudinal VBM studies in alcohol-dependent populations have demonstrated partial gray matter volume recovery following prolonged abstinence, suggesting that neuroplasticity mechanisms may facilitate brain structural restoration (16). However, the extent and rate of recovery vary across individuals, and the factors influencing this variability remain poorly understood.

Given FIR’s potential neuroprotective effects, VBM is an ideal tool to investigate whether FIR therapy accelerates brain recovery by promoting structural reorganization in alcohol-dependent individuals. This study employs longitudinal MRI and VBM combined with cognitive assessment (MoCA) to assess the effects of FIR treatment on gray matter volume recovery in individuals with alcohol dependence in relation to improvements in cognitive functioning related to executive control and memory. By examining changes in key cognitive and affective brain regions, we aim to determine whether FIR treatment promotes cognitive stabilization and structural recovery more than conventional withdrawal treatment alone.

## 2 Materials and methods

### 2.1 Participants

This study recruited 50 male alcohol-dependent patients, aged 18 to 65 years, who were assigned to either a conventional withdrawal group or a FIR intervention group. Allocation to the FIR or withdrawal group was non-randomized and primarily based on logistical considerations (e.g., specific ward of admission) rather than baseline clinical severity. Assignments were assessed by two attending physicians to ensure balanced distributions of age and education levels across groups. This clinical trial was prospectively registered on the National Health Security Information Platform for Medical Research Registration and Archiving System (registration number: MR-34-23-028757) on November 1, 2022. Participants underwent comprehensive clinical assessments, including general data collection, medical history, laboratory tests, and physical examinations. Inclusion criteria were as follows: patients receiving inpatient alcohol withdrawal treatment, aged 18 to 65 years, meeting the ICD-10 diagnostic criteria for Alcohol dependence syndrome, having no significant withdrawal symptoms (CIWA-Ar<7, MMSE≥26), and having consumed alcohol for at least one year before systematic alcohol withdrawal treatment. Exclusion criteria included contraindications to TMS, MRI, or EEG; severe or uncontrolled systemic or psychiatric diseases; other addictive behaviors or substance dependence; and the use of medications affecting alcohol cravings within the past month. The Ethics Committee of the Fourth People’s Hospital of Hefei approved the study (Registration Number: HFSY-IRB-YJ-YJSKT-XQR), and all participants provided informed consent.

### 2.2 Far infrared intervention

Far infrared (FIR) intervention primarily utilizes three types of devices: FIR saunas, FIR ray-emitting equipment, and FIR-emitting ceramics and fabrics (6). Each method delivers therapeutic effects by transmitting radiant heat in the FIR spectrum, which promotes blood circulation, enhances metabolism, and alleviates muscle tension. FIR saunas are widely used in healthcare due to their direct heat conduction and radiation effects, making them effective for pain relief, inflammation reduction, and physical recovery. In this study, we employed the BY-IFRD1TS5A FIR sauna room, developed by Anhui Zhongke Benyuan Information Technology Co., Ltd., which emits FIR wavelengths between 8 and 10 μm. Research indicates that the optimal biological effects of FIR typically occur within the 8–14 μm range, with wavelengths in this spectrum demonstrating the highest efficacy for biological tissues. FIR in this range is primarily absorbed by the superficial skin layers, generating thermal effects that positively influence microcirculation and neural function (6, 17). Consequently, the 8–10 μm wavelength was specifically selected for its alignment with established therapeutic frequencies that maximize biological responses.

**Figure 1:**
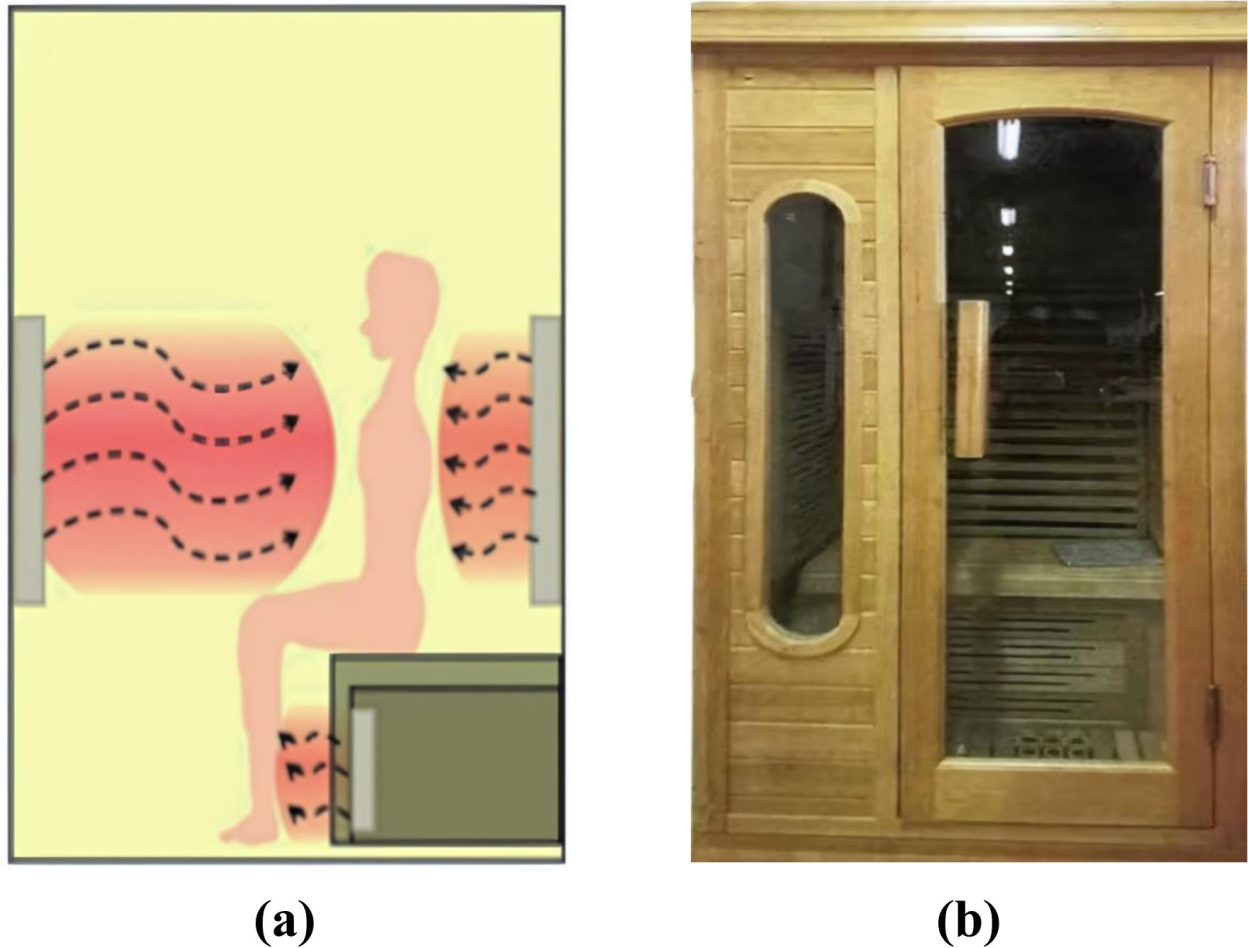
A schematic of the FIR sauna. **(a)** Principle of infrared light intervention. **(b)** Infrared physical therapy room.

Alcohol-dependent patients underwent FIR therapy every other day for a total of five sessions, each lasting 30 minutes. This session duration was chosen because it optimally supports mitochondrial function and ATP generation while minimizing potential adverse effects such as mild dehydration or overheating. Studies indicate that FIR exposure for 10–40 minutes significantly enhances mitochondrial activity, improves ATP production, and stimulates neuroplasticity—key factors in the recovery of alcohol-dependent individuals (10). Additionally, an intermittent treatment schedule, with sessions spaced one day apart, was implemented based on evidence suggesting that intermittent exposure (2–4 times per week) is more effective for maintaining long-term biological benefits than continuous exposure (18). This approach helps prevent cellular tolerance and sustains the activation of neurotrophic factors, such as brain-derived neurotrophic factor (BDNF) (19), which plays a crucial role in neural recovery. By incorporating these scientifically supported parameters, this study aimed to maximize the therapeutic efficacy of FIR therapy while ensuring participant comfort and safety. Patients in the FIR group received the FIR intervention in addition to the standard routine withdrawal treatment received by the control group. All participants included in the final analysis for the FIR group completed the full course of five sessions. **Figure 1** provides a schematic of the FIR sauna.

### 2.3 MRI data acquisition and scale testing

MRI data acquisition was conducted at the Department of Radiology, Fourth People’s Hospital of Hefei, using a 3.0 T GE Discovery MR750 MRI scanner, a high-field device widely employed in clinical and scientific research. The scanner was equipped with an 8-channel birdcage head coil, designed to conform to the shape of the subject’s head and enhance signal reception efficiency. A three-dimensional fast gradient echo T1-weighted sequence was used for structural MRI (sMRI) scanning. The specific parameters were as follows: TR = 8.2 ms, TE = 3.2 ms, matrix size = 256 × 256, with 188 slices acquired, each 1.0 mm thick, and a voxel size of 1 mm×1 mm×1 mm. During data acquisition, participants were instructed to remain awake, keep their eyes closed, lie still on the examination bed, and minimize unnecessary movements. Cotton balls were placed in their ears to reduce noise interference from the MRI machine. All subjects underwent the same MRI sequence scan both before and after the intervention. Specifically, for the FIR intervention group, the post-intervention MRI scan was conducted within 20 days after the final FIR therapy session. For the control group, the second MRI scan was performed approximately one month after routine withdrawal treatment. Although the follow-up window differed slightly between groups, both assessments targeted an early-abstinence period.

The cognitive function and psychological status of the alcohol-dependent patients were assessed using the Montreal Cognitive Assessment (MoCA) (20, 21). We conducted the Montreal Cognitive Assessment (MoCA) test both before the experiment and 4 weeks after the intervention. Montreal Cognitive Assessment, the MOCA included visuospatial and executive function, naming, memory (no score), attention, language, abstraction, delayed recall, and orientation. The maximum total score was 30 points. If the length of education ≤ 12 years, 1 point was added, and < 26 points indicated cognitive impairment. The assessment conducted in this study utilizes the MoCA scale, specifically employing the Mandarin-8.1 version (21). All tests were scored by professionals trained in neuropsychological testing.

### 2.4 MRI image preprocessing

T1-weighted images were processed using the longitudinal pre-processing pipeline from the CAT12 toolbox (Version 2170, Structural Brain Mapping Group, Jena, Germany) based on SPM12 (Institute of Neurology, London, UK) and executed in MATLAB (Version R2020b, The MathWorks, USA) with default settings. The processing involved segmentation into grey matter, white matter, and cerebrospinal fluid using an adaptive mixture model-based method to improve accuracy, especially in cases with low contrast between tissues. Spatial normalization was performed using the Geodesic Shooting template, which aligns individual scans to a common template space using a nonlinear surface-based registration, ensuring high fidelity in maintaining the brain’s geometric structure across timepoints. To ensure consistency across timepoints, the pipeline incorporated a robust longitudinal registration approach that minimizes differences between scans of the same subject collected at different timepoints, using both within-subject and between-subject registrations. Smoothing was performed with an 8 mm full width at half maximum (FWHM) Gaussian kernel to reduce noise and increase signal-to-noise ratio for subsequent analysis. This preprocessing pipeline also includes bias field correction to adjust for inhomogeneities in the magnetic field that might affect the intensity distribution across the images, enhancing the quality and consistency of the final images for longitudinal analysis.CAT12 was also used to estimate total intracranial volume (TIV) for each participant.

### 2.5 Statistical analysis

Statistical analyses of demographic and clinical variables were performed in SPSS 27.0. Normality of data distribution was assessed using the Shapiro-Wilk test, and homogeneity of variance was assessed using Levene’s test. Continuous variables are reported as mean ± standard deviation and were compared using independent-samples t-tests (or Mann–Whitney U tests when assumptions were violated). Categorical variables were compared using chi-square tests.

**Figure 2:**
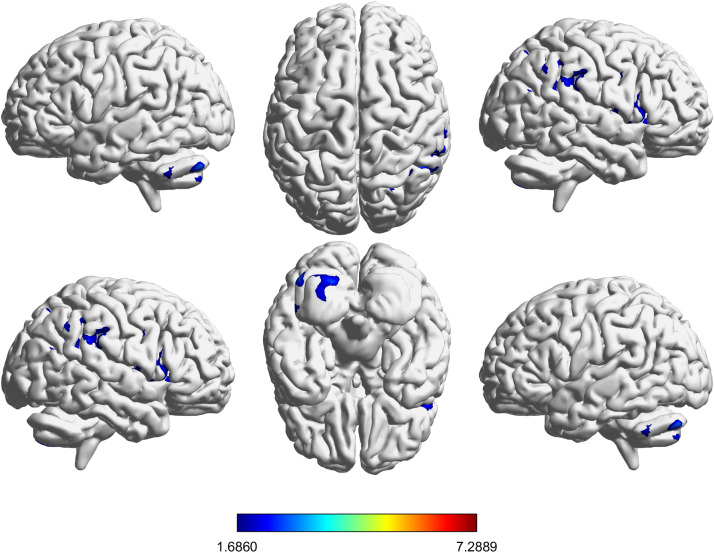
Between-group comparison of longitudinal gray matter volume change, reflecting greater longitudinal GMV increases in the FIR group relative to the withdrawal group (difference-in-differences contrast). Maps are displayed using a voxel-forming threshold of p < 0.001 (uncorrected) and a cluster-level FDR-corrected threshold of p < 0.05 (two-sided). Color bars represent T values.

For voxel-wise VBM analyses in SPM12, within-group longitudinal changes were assessed using paired-sample t-tests (Post *vs*. Pre). To control for inter-individual differences in head size, total intracranial volume (TIV) estimated by CAT12 was included as a covariate of no interest in all second-level voxel-wise general linear models (GLMs). All voxel-wise tests were two-sided. Whole-brain tests were performed in both directions (GMV increases and decreases). Statistical inference used a voxel-forming threshold of p < 0.001 (uncorrected) and cluster-level FDR correction at p < 0.05 (two-sided). Under this predefined threshold, only positive clusters were observed in the contrasts reported here; therefore, figures display positive clusters. Peak T values and MNI coordinates were extracted using xjview. For between-group inference of longitudinal change (**Figure 2**), individual difference maps (Post–Pre) were entered into a second-level two-sample t-test with TIV as a covariate.

To directly evaluate whether FIR provided additional effects beyond routine withdrawal in ROI-based measures, we conducted a mixed-design ANOVA with Group (FIR *vs*. Withdrawal) as a between-subject factor and Time (Pre *vs*. Post) as a within-subject factor, focusing on the Group × Time interaction. Finally, to explore coordinated structural changes, we conducted an exploratory pairwise ROI-based mixed-design ANOVA. This analysis modeled GMV changes in pairs of anatomical regions to detect potential coupled recovery patterns (structural covariance). The significance level for these exploratory interaction effects was set at p < 0.05 (uncorrected) or trend-level p < 0.10, given the hypothesis-generating nature of this analysis.

## 3 Results

### 3.1 Demographic characteristics

This study initially recruited 50 male patients diagnosed with alcohol dependence. However, due to factors such as strong personal requests for discharge, family-related issues, and varying recovery progress, six patients from the FIR group and four from the conventional withdrawal group withdrew from the study, leading to their exclusion. As a result, the final analysis included data from 40 patients. Despite these exclusions, as shown in **Table 1**, the two groups showed no statistically significant differences (P > 0.05) in age, education years, marital status, years of drinking, standard drink, or MoCA scores. Baseline TIV did not differ between groups (Withdrawal: 1446 ± 95 ml; FIR: 1452 ± 98 ml; p = 0.846). In addition, TIV remained stable across time in both groups (Withdrawal: 1444 ± 97 ml; FIR: 1450 ± 101 ml), reducing concern that head-size differences confounded the VBM findings.

**Table 1:** Sample characteristics of patients in the withdrawal and FIR groups.

Variables	Withdrawal (n=21)	FIR (n=19)	P-Value
Age	41.71 ± 11.02	38.00 ± 8.38	0.235
Education years	9.57 ± 3.09	9.37 ± 2.75	0.828
Marital status (married)	13/21	13/19	0.666
Years of drinking	20.4 ± 10.86	17.11 ± 8.57	0.293
Standard drink ^1^	24.29 ± 10.19	25.11 ± 15.78	0.845
MoCA^2^	21.52 ± 4.41	24.00 ± 3.58	0.060
TIV(ml)	1446 ± 95	1452 ± 98	0.846

Data are presented as mean ± standard deviation unless otherwise indicated.

A standard drink refers to approximately 10 grams of pure ethanol according to the World Health Organization (WHO). MoCA, Montreal Cognitive Assessment; TIV, total intracranial volume estimated using the CAT12 toolbox.

### 3.2 Longitudinal MRI changes of routine withdrawal

**Figure 3:**
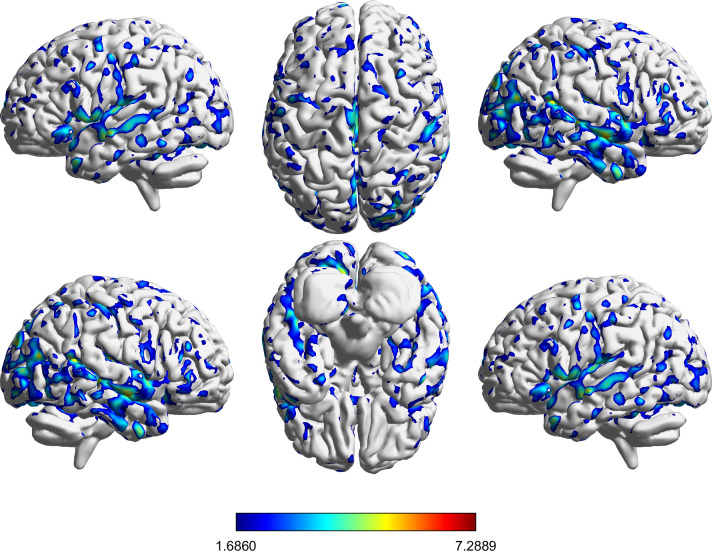
VBM results showing gray matter volume changes in the routine withdrawal group (Post > Pre). Maps are displayed using a voxel-forming threshold of p < 0.001 (uncorrected) and a cluster-level FDR-corrected threshold of p < 0.05 (two-sided). Color bars represent T values.

**Table 2** and **Figure 3** illustrate the enhancement in gray matter volume before and after routine withdrawal. In **Figure 3**, the color scale legend represents the statistical T-values, which reflects the significant changes in gray matter volume. At the predefined threshold (voxel p < 0.001 uncorrected; cluster-level FDR p < 0.05, two-sided), only positive clusters were observed in this contrast; therefore, figures display positive clusters. Notably, the Brodmann area system does not comprehensively encompass all brain regions, particularly subcortical and cerebellar areas. Therefore, for clusters without corresponding Brodmann area annotations, the Automated Anatomical Labeling (AAL) atlas was used as an alternative reference. Additionally, large significant clusters may extend across multiple regions, making it challenging to assign a single Brodmann area to every voxel. Some clusters lack explicit Brodmann area designations because VBM results are reported based on peak voxel significance and cluster extent rather than strict anatomical boundaries. Analysis of **Table 2** and **Figure 3** reveals that significant gray matter alterations primarily occur in key functional brain regions, including the middle temporal gyrus (Temporal_Mid), cingulate gyrus (Cingulum), occipital sulcus (Calcarine), cerebellum (Cerebellum), parietal lobe (Precuneus, Parietal), precuneus (Cuneus), and hippocampus (Hippocampus). These regions are critically involved in cognitive functions, sensory integration, emotional regulation, motor control, and autonomic nervous system functions.

**Table 2:** Areas of gray matter volume enhancement before and after routine withdrawal.

Significant clusters	Brodmann areas	Cluster size	p-value (FDR-corr)	T	MNI_xyz_
Temporal_Mid_R, Temporal_Mid_L, Temporal_Inf_R, Cingulum_Mid_L, Calcarine_L, Temporal_Sup_R, Cerebelum_6_R, Precuneus_R, Lingual_R, Cerebelum_6_L, Insula_R, Precuneus_L, Fusiform_R, Lingual_L, Fusiform_L, Temporal_Inf_L, Occipital_Mid_R, Temporal_Sup_L, Cingulum_Mid_R, Cerebelum_Crus1_L, Insula_L, Cuneus_L, Supp_Motor_Area_L, Frontal_Sup_Medial_L, Occipital_Mid_L, Calcarine_R, Temporal_Pole_Sup_L, Rolandic_Oper_R, Postcentral_L, Cerebelum_4_5_R, Occipital_Sup_R, Supp_Motor_Area_R, Postcentral_R, Frontal_Mid_L, Cerebelum_Crus1_R, Temporal_Pole_Sup_R, Cerebelum_4_5_L, Rolandic_Oper_L, Caudate_L, Frontal_Inf_Orb_L, Parietal_Sup_R, Parietal_Inf_L, Precentral_L, Frontal_Inf_Orb_R, Frontal_Mid_R, Precentral_R, Cuneus_R, Temporal_Pole_Mid_R, ParaHippocampal_R, Frontal_Sup_L, Paracentral_Lobule_L, Cerebelum_9_L, Cerebelum_Crus2_L, Hippocampus_R, Hippocampus_L, Caudate_R, Parietal_Inf_R, Occipital_Sup_L, Cerebelum_8_L, Frontal_Med_Orb_L, Angular_R, Occipital_Inf_R, Thalamus_L, Frontal_Inf_Tri_L, Vermis_6, Cingulum_Ant_L, Frontal_Inf_Tri_R, SupraMarginal_R, Rectus_L, ParaHippocampal_L, Temporal_Pole_Mid_L, Vermis_4_5, Cingulum_Ant_R, Occipital_Inf_L, Frontal_Sup_R, Heschl_L, Thalamus_R, Parietal_Sup_L, Paracentral_Lobule_R, Heschl_R, SupraMarginal_L, Frontal_Inf_Oper_R, Cingulum_Post_L, Frontal_Sup_Medial_R, Cerebelum_9_R, Olfactory_L, Putamen_L, Frontal_Inf_Oper_L, Frontal_Med_Orb_R, Cerebelum_Crus2_R, Olfactory_R, Angular_L, Vermis_9, Frontal_Sup_Orb_L, Cerebelum_8_R, Cerebelum_7b_L, Rectus_R, Frontal_Mid_Orb_L, Vermis_7, Vermis_8, Amygdala_L, Cerebelum_10_L, Pallidum_L, Vermis_10, Frontal_Mid_Orb_R, Cerebelum_10_R, Cingulum_Post_R, Cerebelum_3_R, Vermis_3, Amygdala_R, Frontal_Sup_Orb_R, Cerebelum_3_L, Vermis_1_2, Cerebelum_7b_R, Putamen_R	19, 18, 6, 13, 31, 22, 7, 21, 38, 37, 10, 40, 32, 20, 47, 39, 8, 9, 30, 11, 24, 23, 46, 5, 25, 41, 2, 36, 45, 42, 17, 4, 34, 3, 43, 28, 29, 1, 35, 44, 27, 33	128944	<0.001	7.2889	3 -66 -51

(applies to **Table 2**–**4**): All significant clusters are reported using a voxel-forming threshold of p < 0.001 (uncorrected), followed by cluster-level false discovery rate (FDR) correction at p < 0.05 (two-sided). Anatomical labels are defined using the AAL atlas; Brodmann areas are provided when applicable. Cluster size is reported in voxels. T and MNI (x, y, z) indicate the peak voxel within each cluster. Reported p values correspond to cluster-level FDR-corrected p values. All voxel-wise results were obtained with total intracranial volume (TIV) included as a covariate in second-level models.

### 3.3 Longitudinal MRI changes of far infrared intervention

**Figure 4:**
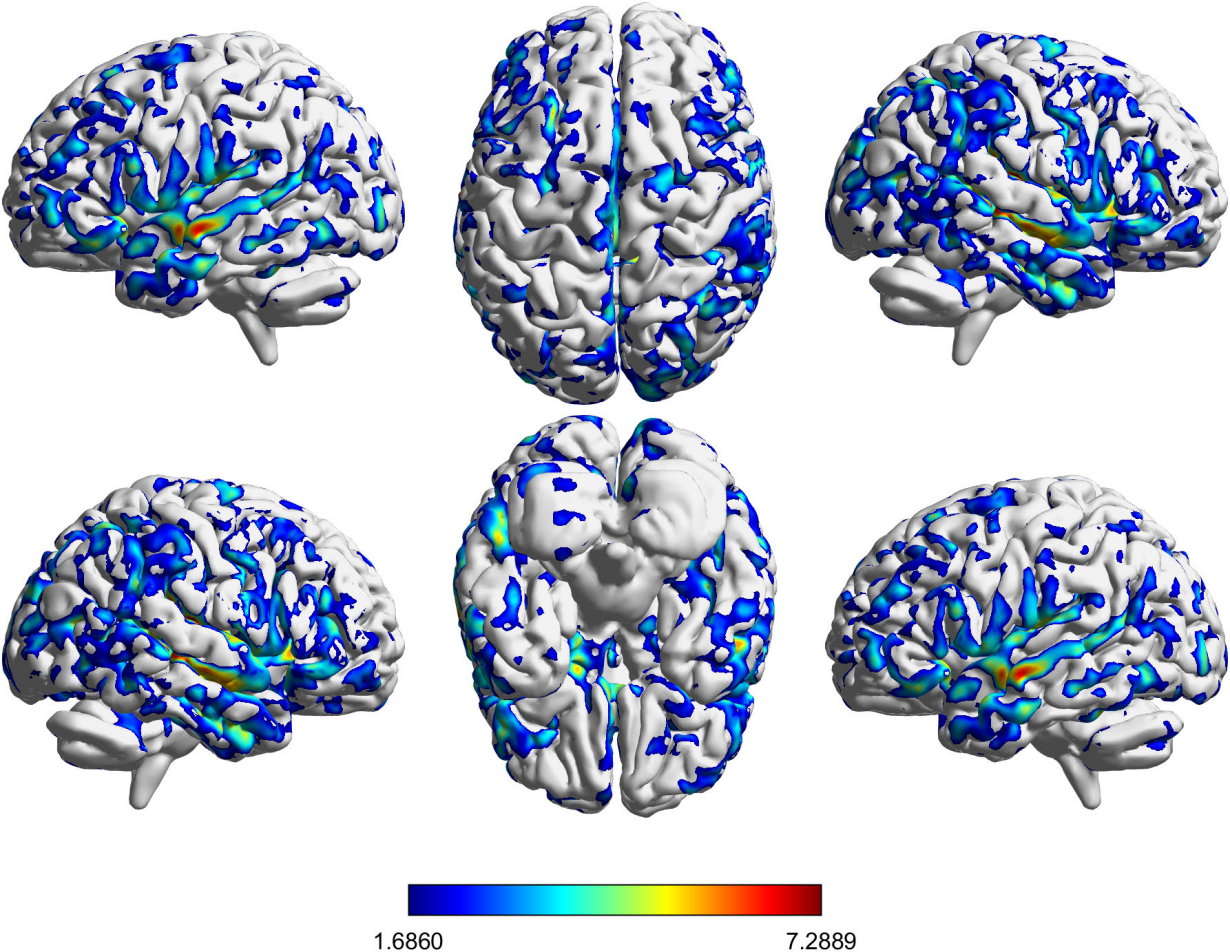
VBM results showing gray matter volume changes in the FIR group (Post > Pre). Maps are displayed using a voxel-forming threshold of p < 0.001 (uncorrected) and a cluster-level FDR-corrected threshold of p < 0.05 (two-sided). Color bars represent T values.

**Table 3** and **Figure 4** illustrate the increase in gray matter volume resulting from the FIR intervention. The results indicate significant alterations in multiple brain regions before and after the intervention. These regions include the temporal lobe, cingulate gyrus, parietal lobe, occipital lobe, and cerebellum. Specifically, substantial changes were observed in the middle temporal gyrus, inferior temporal gyrus, and superior temporal gyrus. Additionally, significant gray matter volume increases were detected in the cingulate gyrus and precuneus. Furthermore, increased gray matter volume was observed in various regions of the cerebellum (e.g., Cerebellum_6, Cerebellum_8, and Cerebellum_Crus). Further analysis revealed particularly pronounced changes in the prefrontal regions, including the middle frontal gyrus, superior frontal gyrus, inferior frontal gyrus (triangular part), and orbitofrontal cortex.

**Table 3:** Areas of gray matter volume enhancement before and after far infrared intervention.

Significant clusters	Brodmann areas	Cluster size	p-value (FDR-corr)	T	MNI_xyz_
Temporal_Mid_R, Temporal_Inf_R, Temporal_Mid_L, Temporal_Sup_R, Cingulum_Mid_L, Precuneus_R, Frontal_Mid_L, Insula_R, Temporal_Inf_L, Lingual_R, Frontal_Mid_R, Fusiform_R, Precuneus_L, Fusiform_L, Cingulum_Mid_R, Calcarine_R, Temporal_Sup_L, Postcentral_R, SupraMarginal_R, Calcarine_L, Occipital_Mid_R, Supp_Motor_Area_L, Cingulum_Ant_L, Frontal_Sup_Medial_L, Precentral_R, Parietal_Sup_R, Frontal_Inf_Tri_L, Lingual_L, Parietal_Inf_R, Insula_L, Frontal_Inf_Tri_R, Cerebelum_6_R, Angular_R, Cuneus_R, Supp_Motor_Area_R, Precentral_L, Cerebelum_Crus1_L, Temporal_Pole_Sup_L, Rolandic_Oper_R, Postcentral_L, Occipital_Sup_R, Frontal_Inf_Oper_R, Cuneus_L, Temporal_Pole_Sup_R, Frontal_Sup_L, Frontal_Inf_Orb_L, Occipital_Mid_L, Parietal_Inf_L, Frontal_Inf_Orb_R, Paracentral_Lobule_L, Frontal_Sup_R, Rolandic_Oper_L, Cerebelum_8_L, Cerebelum_Crus2_L, Rectus_L, Cerebelum_6_L, Caudate_L, Temporal_Pole_Mid_R, Cingulum_Ant_R, Hippocampus_L, Frontal_Sup_Medial_R, Frontal_Inf_Oper_L, Thalamus_L, SupraMarginal_L, Occipital_Sup_L, Parietal_Sup_L, Frontal_Med_Orb_L, Cerebelum_4_5_L, Cerebelum_Crus2_R, Cerebelum_Crus1_R, Frontal_Mid_Orb_R, Occipital_Inf_L, Caudate_R, ParaHippocampal_R, Cerebelum_4_5_R, ParaHippocampal_L, Olfactory_L, Hippocampus_R, Putamen_L, Temporal_Pole_Mid_L, Cerebelum_8_R, Cingulum_Post_L, Paracentral_Lobule_R, Occipital_Inf_R, Heschl_L, Thalamus_R, Heschl_R, Frontal_Mid_Orb_L, Olfactory_R, Cerebelum_7b_L, Frontal_Sup_Orb_L, Putamen_R, Frontal_Sup_Orb_R, Cerebelum_9_L, Cingulum_Post_R, Rectus_R, Frontal_Med_Orb_R, Angular_L, Vermis_6, Cerebelum_7b_R, Vermis_7, Vermis_4_5, Cerebelum_3_R, Amygdala_L, Cerebelum_9_R, Vermis_8, Pallidum_R, Cerebelum_10_R, Amygdala_R, Vermis_1_2, Pallidum_L, Cerebelum_10_L, Vermis_3, Vermis_10, Cerebelum_3_L	6, 19, 7, 40, 18, 21, 22, 31, 13, 10, 9, 38, 20, 32, 37, 8, 11, 39, 47, 24, 46, 45, 4, 17, 25, 2, 5, 30, 23, 41, 44, 3, 34, 42, 36, 43, 28, 1, 29, 27, 35, 33	179188	<0.001	8.097	52 -21 14

### 3.4 Far infrared intervention enhanced MRI changes compared with routine withdrawal

**Table 4** and **Figure 2** present the brain regions with increased gray matter volume following FIR intervention compared to conventional withdrawal. The findings suggest that FIR intervention was associated with greater longitudinal GMV increases in multiple brain regions compared with routine withdrawal. First, in the cerebellar regions (e.g., Cerebellum_Crus1_L, Cerebellum_Crus2_L, Cerebellum_8_L, and Cerebellum_7b_L), a substantial increase in gray matter volume (cluster size: 2591) was observed. Second, significant increases in gray matter volume (cluster size: 2777) were found in the prefrontal cortex and insula (e.g., Frontal_Inf_Oper_R, Precentral_R, Postcentral_R, Temporal_Sup_R). Finally, the largest cluster (cluster size: 4054) was identified in the right temporal, parietal, and occipital lobes (e.g., SupraMarginal_R, Parietal_Inf_R, Temporal_Mid_R). Notably, **Figure 2** shows relatively smaller changes compared to **Figure 4** because it reflects regions showing greater longitudinal gray matter volume increases in the FIR group relative to the withdrawal group, rather than the overall pre- and post-intervention difference within a single group. In addition to the voxel-wise difference-in-differences analysis, results from the exploratory pairwise ROI-based analysis (examining coupled structural recovery) are summarized in **Supplementary Table 1**. These findings are presented as complementary evidence to the voxel-wise results.

**Table 4:** Areas of gray matter volume enhancement following FIR intervention compared to routine withdrawal.

Significant clusters	Brodmann areas	Cluster size	p-value (FDR-corr)	T	MNI_xyz_
Cerebelum_Crus1_L, Cerebelum_Crus2_L, Cerebelum_8_L, Cerebelum_7b_L		2591	<0.001	4.0939	-40 -75 -33
Frontal_Inf_Oper_R, Frontal_Inf_Tri_R, Rolandic_Oper_R, Precentral_R, Insula_R, Postcentral_R, Temporal_Sup_R, Frontal_Mid_R, Frontal_Inf_Orb_R, Heschl_R, Temporal_Pole_Sup_R	45, 44, 9, 6, 47, 22, 13, 46, 4, 21, 43	2777	<0.001	3.5936	52 9 3
SupraMarginal_R, Parietal_Inf_R, Angular_R, Temporal_Mid_R, Temporal_Inf_R, Parietal_Sup_R, Temporal_Sup_R, Postcentral_R, Rolandic_Oper_R, Fusiform_R, Occipital_Mid_R, Occipital_Sup_R	40, 7, 37, 2, 39, 20, 19, 3, 13, 22, 1, 4, 21	4054	<0.001	3.2871	54 -54 39

### 3.5 Effects of far infrared intervention relative to conventional withdrawal on patients’ cognitive functioning

The baseline MoCA scale scores have been presented in **Table 1**. After routine withdrawal of medication, we re-administered the scale tests. As shown in **Table 5**, the mean MoCA scores of the FIR group were consistently higher than the control group scores. The difference in mean scores between the two groups was 1.130, whereas the usual MoCA minimum clinically important difference (MCID) is 1–2 points. An independent samples t-test showed this difference to be close to significance (p = 0.052), and Cohen’s d = 0.635 indicated a moderate effect. Given that the FIR group data did not conform to a normal distribution, we further performed a nonparametric Mann-Whitney U test, which yielded a U value of 270.500, with an asymptotic significance of p = 0.051.

**Table 5:** Intergroup analysis of MoCA scale scores after drug discontinuation.

Variable	Groups (M ± SD)		Contrast	Statistic	P-Value	Cohen’s d
MoCA	Withdrawal (n=21)	FIR (n=19)	Difference in Means	1.130		
	27.24 ± 1.868	28.37 ± 1.674	Independent Samples t-Test	2.007	0.052	0.635
			Mann-Whitney U Test	270.500	0.051	

## 4 Discussion

### 4.1 Interpretation of findings

This study examined the effects of FIR therapy on brain structure in patients with Alcohol dependence and compared its neurorestorative effects with those of routine withdrawal. The results indicate that FIR therapy was associated with increased gray matter volume across multiple key brain regions, particularly in the prefrontal cortex, insula, and cerebellum. Because no direct physiological or molecular biomarkers (e.g., cerebral blood flow measures, BDNF, inflammatory markers) were collected, mechanistic interpretations should be considered speculative. This finding suggests that FIR therapy may support recovery-related processes, potentially via systemic hemodynamic and stress-response pathways. In the conventional alcohol abstinence treatment group, patients exhibited increased gray matter volume in several brain regions, including the middle temporal gyrus, cingulate gyrus, occipital sulcus, cerebellum, parietal lobe, cuneus, and hippocampus following treatment. These regions are primarily involved in cognitive function, emotion regulation, and sensory-motor integration, indicating that alcohol abstinence treatment contributes to the recovery of certain brain structures (22). However, the degree and rate of recovery varied among individuals.

Compared to those who underwent only alcohol abstinence treatment, patients who received FIR therapy showed more pronounced GMV increases in several regions. These patterns may be associated with stronger recovery-related structural changes, although causal inference cannot be established in this non-randomized pilot study. The cerebellum plays a crucial role in motor control and cognitive function, and previous studies have shown that chronic alcoholism reduces cerebellar gray matter volume, impairing motor coordination and cognitive abilities (23, 24). Our findings suggest that FIR therapy may be associated with greater cerebellar GMV increases, potentially via systemic heat-related stress responses and related neurotrophic pathways; these mechanisms require confirmation using direct biomarkers. Furthermore, the prefrontal cortex is essential for executive function, decision-making, and emotional regulation, while the insula is involved in self-awareness, sensory perception, and emotional experience. Prior research has demonstrated that Alcohol dependence significantly reduces gray matter volume in the prefrontal cortex and insula, which correlates with increased impulsivity and impaired decision-making (25, 26). The present study suggests that FIR therapy may be associated with greater GMV increases in these regions, which could be relevant to executive and affective functions, but should be interpreted cautiously given the study design. The temporal-parietal region primarily contributes to language processing, spatial cognition, and memory function. Studies have shown that Alcohol dependence leads to reduced gray matter volume in this region, subsequently affecting cognitive flexibility and information processing (23). This study observed greater GMV increases in temporal–parietal regions in the FIR group, which may be relevant to cognitive functions such as memory and language, warranting further investigation.

In addition to these structural findings, the results of our cognitive assessment provide some insights, such as consistently higher MoCA scores in the FIR group than in the control group at baseline and after routine withdrawal, with the difference converging to statistical significance and showing a medium effect size. Although independent samples t-tests (p=0.052) and Mann-Whitney U-tests (p=0.051) did not reach conventional statistical significance, the consistent positive trend and effect size of 0.635 suggest a consistent positive trend with a moderate effect size, warranting further investigation.

This pilot study provides preliminary evidence that FIR therapy may be associated with greater GMV increases in regions related to executive function, cognition, and motor control. Although the precise mechanisms of FIR therapy require further investigation, its non-invasive nature suggests potential clinical relevance that warrants confirmation in larger randomized studies. Future research should incorporate behavioral assessments and long-term follow-up to better elucidate the sustained effects and underlying mechanisms of FIR therapy in the neurological recovery of patients with Alcohol dependence.

### 4.2 Limitations and future directions

Although this study provides preliminary evidence to support the potential benefits of FIR interventions for neurological recovery in patients with alcohol dependence, several limitations must be addressed in future studies.

First, the sample size of this study was relatively small, containing only 40 subjects. Future studies should expand the sample size and design more rigorous randomized controlled trials (RCTs) to clarify the true effects of FIR interventions.

Second, while within-group voxel-wise paired t-tests were used to characterize longitudinal changes, we additionally conducted an explicit Group × Time interaction analysis for ROI-based measures to directly assess differential trajectories between groups. Nevertheless, the voxel-wise between-group inference should still be interpreted cautiously given the modest sample size and the non-randomized design; future studies with larger samples should implement fully powered mixed-effects or factorial models at the whole-brain level.

Third, although the baseline MoCA scores did not differ significantly between groups (p = 0.060), the FIR group exhibited numerically higher scores. We cannot rule out the possibility that pre-existing baseline differences contributed partially to the post-intervention outcomes. Future randomized controlled trials (RCTs) with strict stratification are needed to eliminate this potential confounder.

Fourth, this study relied primarily on structural MRI (VBM) and the MoCA scale to assess the effects of the FIR intervention. However, structural MRI only reflects changes in gray matter volume and does not directly capture functional changes, such as neural network plasticity or dynamic changes in neural activity. Regarding the cognitive assessment, while the MoCA is a sensitive tool for detecting mild cognitive impairment, it may have ceiling effects or limited sensitivity for specific cognitive deficits associated with alcohol dependence. Therefore, future studies should integrate multimodal imaging techniques, including functional MRI (fMRI), magnetic resonance spectroscopy (MRS), and diffusion tensor imaging (DTI), with a more comprehensive neuropsychological battery targeting executive function and impulsivity to provide a more comprehensive understanding of patient brain structure, function, and cognitive improvement.

Finally, the far infrared irradiation regimen used in this study was based on the existing literature, but systematic studies on optimal dosage parameters (e.g., intensity, duration, and frequency of irradiation) are still insufficient, whereas there may be differences in individual responses to far infrared interventions. In addition, the short follow-up period of this study failed to assess the long-term effects of far infrared intervention. Therefore, future studies should further explore the optimal parameters of individualized far infrared interventions and incorporate longer follow-up periods (e.g., six months to more than one year) to determine whether far infrared therapies provide lasting neuroprotective benefits and to assess their impact on alcohol dependence relapse rates.

## 5 Conclusion

This study investigated the effects of FIR intervention on brain structure in patients with Alcohol dependence. FIR intervention was associated with greater GMV increases in key regions, particularly the prefrontal cortex, insula, and cerebellum. Compared to conventional alcohol abstinence treatment, FIR may be associated with additional structural recovery, potentially via systemic hemodynamic and stress-response pathways; however, these mechanisms require confirmation in studies incorporating direct biomarkers. The results also revealed that while conventional alcohol abstinence treatment led to partial restoration of gray matter volume in brain regions associated with cognition and emotion regulation, the degree of recovery varied among individuals. Notably, the FIR intervention group exhibited greater GMV increases, particularly in regions linked to executive function, emotion regulation, and motor coordination. Although this study provides preliminary evidence supporting FIR’s role in neural recovery, it is limited by factors such as a small sample size, the absence of a placebo control, a lack of multimodal imaging analysis, and insufficient long-term follow-up. Future research should incorporate larger sample sizes, randomized controlled trials, and extended follow-up periods to further validate FIR’s clinical effectiveness and underlying mechanisms, thereby establishing a scientific foundation for its application in Alcohol dependence treatment.

## Data Availability

The datasets presented in this article are not readily available because There are ethical, legal, or privacy-related concerns with sharing the data. Requests to access the datasets should be directed to gwx0306@stu.ahau.edu.cn.
