# Supplementary material for: Far infrared intervention on brain changes in patients with alcohol dependence: a pilot longitudinal MRI study
Source: Front Psychiatry. 2026 Mar 9;17:1759791. doi: 10.3389/fpsyt.2026.1759791 (PMC13006328; doi:10.3389/fpsyt.2026.1759791)
Supplement: Supplementary file 1 [file Table1.docx]

# Supplementary Material

Supplementary Table S1. Selected exploratory pairwise ROI-based mixed-design ANOVA results for Group × Time interaction effects.

| ROI A | ROI B | F (Group × Time) | p-value |
| --- | --- | --- | --- |
| ROI 19 | ROI 21 | 8.489 | 0.004 |
| ROI 21 | ROI 19 | 8.489 | 0.004 |
| ROI 19 | ROI 18 | 6.878 | 0.010 |
| ROI 18 | ROI 19 | 6.878 | 0.010 |
| ROI 10 | ROI 08 | 5.071 | 0.026 |
| ROI 08 | ROI 10 | 5.071 | 0.026 |
| ROI 11 | ROI 19 | 4.854 | 0.030 |
| ROI 19 | ROI 11 | 4.854 | 0.030 |
| ROI 18 | ROI 11 | 4.567 | 0.035 |
| ROI 11 | ROI 18 | 4.567 | 0.035 |
| ROI 12 | ROI 04 | 4.213 | 0.043 |
| ROI 04 | ROI 12 | 4.213 | 0.043 |
| ROI 04 | ROI 20 | 4.136 | 0.044 |
| ROI 20 | ROI 04 | 4.136 | 0.044 |
| ROI 21 | ROI 20 | 4.044 | 0.047 |
| ROI 20 | ROI 21 | 4.044 | 0.047 |
| ROI 11 | ROI 10 | 4.028 | 0.047 |
| ROI 10 | ROI 11 | 4.028 | 0.047 |
| ROI 04 | ROI 16 | 3.867 | 0.052 |
| ROI 16 | ROI 04 | 3.867 | 0.052 |
| ROI 10 | ROI 13 | 3.815 | 0.053 |
| ROI 13 | ROI 10 | 3.815 | 0.053 |
| ROI 19 | ROI 22 | 3.776 | 0.055 |
| ROI 22 | ROI 19 | 3.776 | 0.055 |
| ROI 14 | ROI 19 | 3.535 | 0.063 |
| ROI 19 | ROI 14 | 3.535 | 0.063 |
| ROI 09 | ROI 21 | 3.453 | 0.066 |
| ROI 21 | ROI 09 | 3.453 | 0.066 |
| ROI 13 | ROI 05 | 3.203 | 0.076 |
| ROI 05 | ROI 13 | 3.203 | 0.076 |
| ROI 12 | ROI 10 | 3.112 | 0.081 |
| ROI 10 | ROI 12 | 3.112 | 0.081 |
| ROI 24 | ROI 11 | 3.064 | 0.083 |
| ROI 11 | ROI 24 | 3.064 | 0.083 |
| ROI 21 | ROI 14 | 2.905 | 0.091 |
| ROI 14 | ROI 21 | 2.905 | 0.091 |
| ROI 09 | ROI 18 | 2.857 | 0.094 |
| ROI 18 | ROI 09 | 2.857 | 0.094 |
| ROI 19 | ROI 09 | 2.810 | 0.097 |
| ROI 09 | ROI 19 | 2.810 | 0.097 |

Note:

This table reports selected results from an exploratory pairwise ROI-based mixed-design ANOVA examining Group × Time interaction effects on regional gray matter volume (GMV). Group (FIR vs. Withdrawal) was treated as a between-subject factor and Time (Pre vs. Post) as a within-subject factor.

ROI-pair analyses were performed by jointly modeling GMV changes in pairs of predefined ROIs in order to explore whether longitudinal intervention-related effects exhibited coordinated or regionally coupled patterns across anatomically or functionally related regions. In this context, “Seed ROI” and “Comparison ROI” denote the two regions included in each pairwise model, rather than directional or connectivity-based relationships.

Only ROI pairs showing significant or trend-level Group × Time interaction effects (p < 0.10) are reported. These analyses were conducted as supplementary, hypothesis-generating examinations to complement the voxel-wise VBM results presented in the main text and should be interpreted cautiously given the limited sample size. No correction for multiple comparisons was applied in these exploratory ROI-pair analyses..
